# Characterization of an MR‐compatible motion platform for quality assurance of motion‐compensated treatments on the 1.5 T MR‐linac

**DOI:** 10.1002/mp.17632

**Published:** 2025-01-31

**Authors:** Stijn Oolbekkink, Pim T. S. Borman, Jochem W. H. Wolthaus, Bram van Asselen, Astrid L. H. M. W. van Lier, Stephanie Dunn, Grant R. Koenig, Nick Hartman, Niusha Kheirkhah, Bas W. Raaymakers, Martin F. Fast

**Affiliations:** ^1^ Department of Radiotherapy University Medical Center Utrecht Utrecht The Netherlands; ^2^ IBA QUASAR, Modus Medical Devices Inc. London Ontario Canada

**Keywords:** motion‐included dosimetry, MR‐linac, quality assurance

## Abstract

**Background:**

Novel motion‐compensated treatment techniques on the MR‐linac can address adverse intra‐fraction motion effects. These techniques involve beam gating or intra‐fraction adaptations of the treatment plan based on real‐time magnetic resonance imaging (MRI) performed during treatment. For quality assurance (QA) of these workflows, a multi‐purpose motion platform is desirable. This platform should accommodate various phantoms, enabling multiple QA workflows.

**Purpose:**

This study aims to evaluate the new IBA QUASAR Motion MR Platform for use in the 1.5 T MR‐linac.

**Methods:**

The motion platform was assessed for several magnetic resonance (MR) characteristics, including spurious noise generation and B0&B1 homogeneity. In addition, the motion platform's motion accuracy and beam attenuation were assessed. An application was shown with a ScandiDos Delta4 Phantom+ MR demonstrating patient‐specific plan QA of gated treatments using time‐resolved dosimetry that includes motion based on a patient's respiratory motion trace.

**Results:**

All MR characterization measurements were within the set tolerances for MRI QA. The motion platform motion accuracy showed excellent agreement with the reference, with a standard deviation of the amplitude of 0.01  mm (20 kg load) for the motor's self‐estimated positions and 0.22 mm (no load) for the images acquired with the electronic portal imager. Beam attenuation was found to be 11.8%. The combination of the motion platform and Delta4 demonstrated motion‐included dosimetry at high temporal and spatial resolutions. Motion influenced the measured dose in non‐gated treatments by up to −20.1%, while gated deliveries showed differences of up to −1.7% for selected diodes.

**Conclusion:**

The motion platform was found to be usable in a 1.5 T magnetic field, and for all MR characterization experiments, no influence from the motion platform was observed. This motion platform enables to perform motion‐included QA, with a measurement device of choice.

## 1 INTRODUCTION

The combination of a magnetic resonance imaging (MRI) scanner and linear accelerator, the MR‐linac, allows for adaptive workflows that correct for inter‐fraction anatomical changes, resulting in better tumor coverage.^1^, ^2^, ^3^, ^4^, ^5^, ^6^ With novel motion‐compensating treatment techniques, such as the recently introduced comprehensive motion manager (CMM) system designed for the 1.5 T Unity MR‐linac (Elekta AB, Stockholm, Sweden), new workflows can be performed to mitigate adverse intra‐fraction motion effects.^7^, ^8^, ^9^

To simulate anatomical motion and perform quality assurance (QA) for motion‐compensated workflows, a multi‐purpose motion platform is desired and should be compatible with various phantoms and dosimeters. This platform needs to be unaffected by the magnetic field and capable of moving heavy loads with high accuracy. Additionally, it should allow for artifact‐free scanning of these phantoms to facilitate QA of magnetic resonance (MR)‐guided workflows. Potentially, the new IBA QUASAR Motion MR Platform (IBA QUASAR, Modus Medical Devices inc., London, Ontario, Canada) (herein referred to as motion platform) meets these requirements.

The study presents an evaluation of the IBA QUASAR Motion MR Platform, which assesses the MR characteristics, beam attenuation and motion accuracy for use in the 1.5 T MR‐linac. An application was demonstrated using a combination of the motion platform and a Delta4 Phantom+ MR (ScandiDos AB, Uppsala, Sweden) (Delta4).^10^ This setup enables high spatial and temporal dose measurements, facilitating motion‐included, time‐resolved dosimetry for patient‐specific plan QA of gated treatment deliveries.

## 2 MATERIALS AND METHODS

### 2.1 IBA QUASAR Motion MR Platform

All measurements were performed on a 1.5 T MR‐linac using the motion platform. The standard motion platform has a usable surface area of 50.4 cm × 31.1 cm, composed of two 1.9 cm thick PMMA slabs separated by bearings, with feet attached to the lower slab, resulting in a total height of 5.2 cm. The upper slab is displaced in 1D with a motor to simulate various motions, including respiratory traces, and has reference markers for positioning. The motion traces are freely programmable, allowing for customized movement of the motion platform. During measurements, the shielded cable connecting the motion platform to the control unit (located outside the treatment room) passed through the waveguide; the shield was grounded via the RF‐cage.

**FIGURE 1:**
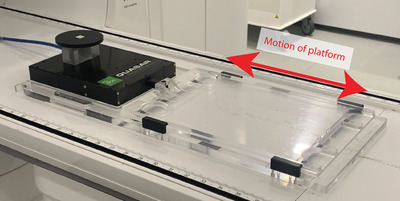
The IBA QUASAR Motion MR Platform with the custom sliding platform in the 1.5 T MR‐linac.

For dosimetric experiments, the motion platform used in this study (see Figure 1) featured a customized upper slab with milled cavities for the feet of a Delta4. Furthermore, the feet of the motion platform were removed, lowering its height to 3.9 cm reducing the distance of the Delta4's central diode to 1.6 cm above the linac's isocenter. This allowed the Delta4 to be recessed at a height closer to its intended position, which is with its central diode at the linac's isocenter. To prevent any sliding motion between the motion platform and the Delta4, rubber traction mats were placed under the Delta4's feet.

### 2.2 Characterization measurements

Three types of characteristics were evaluated in this study. First the MR characteristics in terms of B0&B1 influence, spurious noise generation and image quality were assessed.^11^, ^12^ Secondly, the motion accuracy of the motion platform was evaluated, and lastly the beam attenuation due to the PMMA slabs.

**FIGURE 2:**
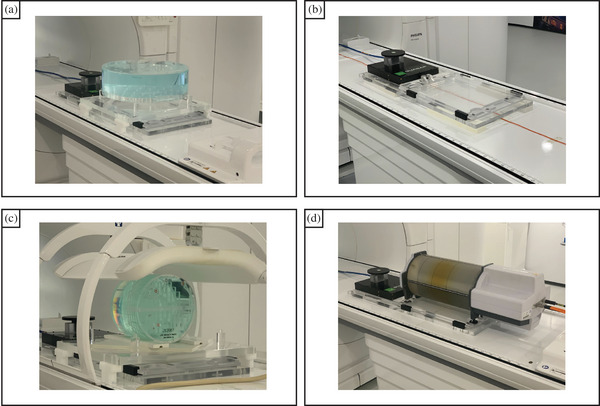
Measurement setups used: (a) The B0&B1 measurement setup. (b) The spurious noise measurement setup. (c) The ACR phantom measurement setup. (d) The combination of the motion platform and a Delta4 phantom. ACR, American College of Radiologist.

The influence of the motion platform on the B0&B1 field homogeneity (see supplementary S1 for scan parameters) was assessed using a Philips body phantom (Philips Healthcare, Best, the Netherlands). The materials of the motion platform might affect the local magnetic field (B0 influence), which in turn would impact geometrical accuracy and image quality. Additionally, the RF path (B1 influence) may be influenced by the materials of the motion platform. To evaluate the potential impact, three different setups were tested: without the motion platform (reference), with the powered motion platform but stationary, and with the slab moving sinusoidally (A = 20 mm, T = 15 bpm). The body phantom was elevated 11 cm (measured from the posterior side of the body phantom) above the motion platform (see Figure 2a) to allow slab movement without moving the body phantom, and removal of the motion platform for the reference measurements. The B0 inhomogeneity in ppm was measured by a dual echo B0 mapping sequence, while the influence of the platform on the flip angle homogeneity, defined as the quantity reaching the desired flip angle, was derived from the dual flip angle B1 measurement. Analysis for both the B0 and B1 measurements was performed on the slice closest to the motion platform. For both the B0 and B1 measurements, the mean value, the standard deviation within the slice, and the range covering the 1st to 99th percentile were calculated. Each measurement was performed twice and the results were averaged.

Spurious noise generated by electrical devices can lead to artifacts in the acquired MR image (e.g., zipper artifacts). Therefore, the potential spurious noise from the motion platform's motor was evaluated in various configurations (powered on and stationary, or moving) using a vendor‐specific test (Philips SupportConnect 10.0). Dot, line, and band spurious noise, along with spurious noise density, were evaluated. A sinusoidal motion pattern with a 20 mm amplitude and a frequency of 15 bpm was used during the measurement involving the motion platform moving. The motion platform was elevated 2 cm from the treatment couch in the anterior direction (see Figure 2b), allowing a 3‐m power cord to be positioned underneath which was used as an antenna. This setup ensured that the motion platform could be removed without interfering with the power cable. The reference measurements were performed without the motion platform in the bore. All measurements were performed twice.

Standardized tests using the American College of Radiologist (ACR) phantom were performed to assess the influence of the (moving) motion platform on the slice thickness accuracy, slice position accuracy, geometric accuracy, high‐contrast spatial resolution, image intensity uniformity and percent‐signal ghosting tests.^13^, ^14^ The ACR phantom setup was elevated (see Figure 2c) 8 cm above the motion platform, allowing the motion platform's upper slab to move freely without moving the ACR phantom, and the motion platform to be removed without interfering with the ACR phantom's position. A sinusoidal motion pattern with a 20 mm amplitude and a 15 bpm frequency was used during the measurement involving the motion platform. Measurements were performed with and without the motion platform moving. As a reference, measurements were performed without the motion platform. Each measurement was performed twice.

Motion accuracy during continuous platform movement was assessed for various motion frequencies (5, 10, 15, 30, and 60 bpm), each evaluated under different loads (0, 10, 20, and 30 kg) resulting in 20 permutations. This was done to ensure the platform can handle the weight of the Delta4, which is 25 kg. The intended sinusoidal motion with an amplitude of 20 mm, defined by the software, was compared to reference motion signals using two methods: a log file recording the motor's self‐estimated position over time, and electronic portal imaging device (EPID) images acquired at 30 Hz tracking a marker placed on the motion platform during beam‐on. The motion was evaluated over a period of ≈ 45 s.

Beam attenuation due to the motion platform's PMMA slabs were determined using a Farmer‐type ionization chamber (PTW Dosimetry GmbH, Freiburg, Germany) (TW30013, SN009627), which was mounted in a RW3 slab filled with water.^15^ Beams were delivered from gantry angle 0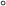, with a field aperture of 10 cm × 10 cm and 100 MU per beam. Each measurement, with and without motion platform in the beam path, was performed five times and averaged. The combined build‐up of the RW3 plates to the center of the ionization chamber was 6.3 cm. Before each measurement, the central position of the ionization chamber was verified using EPID images. Additionally, beam attenuation due to the motion platform was modeled in the Monaco v.6.2.1.0 (Elekta AB, Stockholm, Sweden) treatment planning system (TPS), using a relative electron density of 1.159 for the PMMA slabs with a 1 × 1 × 1 ${\mathrm{mm}}^{3}$ grid and a statistical uncertainty of 0.5% in the dose calculation.

### 2.3 Time‐resolved and motion‐included plan QA

The combination of the motion platform and Delta4 allowed for high spatial and temporal, motion‐included, time‐resolved dosimetry, enabling the evaluation of the effect of respiratory motion on the delivered dose.^16^ To demonstrate this combination for time‐resolved, motion‐included plan QA, several measurements were performed using a Unity MR‐linac in research mode, allowing for gating based on the platform's self‐reported position. Intra‐fraction motion was simulated by moving the Delta4, mounted on the motion platform (Figure 2d), according to a patient‐derived respiratory trace (${\bar{A}}_{peak-to-peak}$ = 18 mm, $\bar{T}$ = 5 s). A research version of the Delta4 software was employed for time‐resolved dosimetry, providing dose readouts from the 1069 diodes every 25 ms.

Three clinical pancreas SBRT plans were recalculated in the Monaco TPS on CT scans of the measurement setup. Measurements including motion were compared to reference measurements, in which the setup did not move. Gated measurements with a duty cycle of ≈50% and non‐gated scenarios with setup movement were evaluated. The dose of two diode locations were evaluated: the diode receiving the highest dose in the reference plan ($D_{max\;dose}^{diode}$), and a diode located near the ≈80% measured isodose line ($D_{80\%\;isodose}^{diode}$). For cumulative comparison of the measured dose to the calculated TPS dose, a global gamma analysis on all diodes (2%/2 mm, threshold ≥ 20% of the isocenter dose) was performed.^17^

## 3 RESULTS

### 3.1 Characterization measurements

No changes in B0 were observed (see Table 1) when comparing scans with the motion platform (with and without motion) with the reference measurements. A similar result for the B1 influence was obtained. The results with the motion platform showed similar flip angle efficiencies, compared to the reference.

**TABLE 1:** The obtained results for the B0 disturbance and B1 flip angle efficiency for the various measurement setups.

	B0 homogeneity			B1 homogeneity		
Setup	Mean (ppm)	σ (ppm)	$p_{99}-p_{1}$ (ppm)	Mean (%)	σ (%)	$p_{99}-p_{1}$ (%)
Reference	0.3	0.4	2.0	82.4	9.8	38.0
Stationary	0.3	0.4	2.0	82.6	9.7	37.4
Moving	0.3	0.4	2.0	82.2	9.8	37.9

*Note*: The σ represents one standard deviation. The difference between the 99th and 1st percentile is shown in the $p_{99}-p_{1}$ column.

All spurious noise tests of the motion platform, with the platform either stationary or moving, met the set criteria by the software across all measurements, and values found were similar to the reference measurements.

All ACR measurements met set tolerances (see supplementary S2), with the exception of the high‐contrast spatial resolution tests. The high‐contrast spatial resolution measurements passed for the 1.0 mm and 1.1 mm resolutions in both $T_{1}$ and $T_{2}$ weighted images for all measurements. The 0.9 mm resolution test never passed (with or without the motion platform present), which is a common occurrence for this specific test on the MR‐linacs.^18^

Motion accuracy was assessed by comparing the intended motion with both the motor's self‐estimated positions and EPID images. The mean correlation ratios for the 20 frequency and load permutations were 1.00 for both the motor's self‐estimated positions and the EPID images, compared to the intended motion pattern. The lowest correlation ratios observed were 0.99 for motor‐reported positions and 0.98 for EPID images, with these values found in different measurements. For these measurements, the standard deviations of the amplitude were 0.01 mm (with a 20 kg load) for the motor's self‐estimated positions and 0.22 mm (with no load) for EPID images, and were for the 60 bpm measurement.

Finally, the beam attenuation due to the motion platform was measured to be 11.8%. The simulated attenuation in the Monaco TPS was found to be 12.1%, and showed good agreement with the measurement.

### 3.2 Time‐resolved and motion‐included plan QA

**FIGURE 3:**
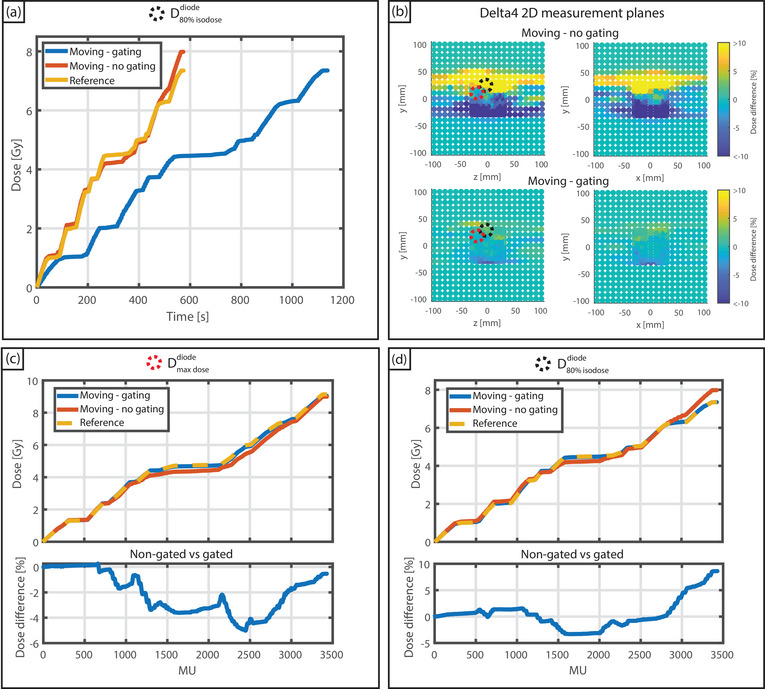
(a) Time‐resolved cumulative dose readout for one of the measurements performed. The dashed red line indicates when the reference measurement finished. (b) Dose differences of the moving non‐gated, and moving gated measurements with respect to the reference measurement. The effect of gated and non‐gated deliveries on the dose blurring for: $D_{max\;dose}^{diode}$ (c) and $D_{80\%\;isodose}^{diode}$ (d). Dose differences are with respect to the reference measurement and were normalized to the measured dose of the reference.

One of the three measured plans is plotted, showing the dose evolution over time for the reference, gated, and non‐gated conditions (Figure 3a). The gated, non‐gated, and reference measurements were 7.35, 7.98, and 7.35 Gy for the diode located at the 80% isodose line, respectively, showing a difference of 0.63 Gy when comparing the non‐gated measurement to both the gated and reference measurements. The gated and reference measurement showed excellent agreement. Differences of the cumulative dose for both the gated and non‐gated measurement relative to the reference are shown in Figure 3b, showing the influence of motion on the delivered dose. Figures 3c and 3d display accumulated dose versus delivered MU, which highlights the potential of this setup to measure the influence of motion on the dose relative to the reference, non moving condition. For this measurement, the gated delivery showed minor differences, with maximum variations of 1.2% and 0.7% for $D_{max\;dose}^{diode}$ and $D_{80\%\;isodose}^{diode}$ during the measurements, respectively, relative to the reference. For non‐gated delivery (with motion) differences were 6.0% and 8.7% for $D_{max\;dose}^{diode}$ and $D_{80\%\;isodose}^{diode}$ during the measurements, respectively, relative to the reference. The results of all measured plans are shown in Table 2, indicating similar outcomes.

**TABLE 2:** The range of differences for both $D_{max\;dose}^{diode}$ and $D_{80\%\;isodose}^{diode}$, and the range of gamma analysis pass rates relative to the TPS dose for the three measured plans.

Setup	Max. dose difference $D_{max\;dose}^{diode}$ (%)	Max. dose difference $D_{80\%\;isodose}^{diode}$ (%)	γ pass rate (%)
Reference	−	−	[98.4, 99.5]
Moving ‐ gating	[−1.7, 0.3]	[−2.5, 0.7]	[96.2, 99.1]
Moving ‐ no gating	[−9.4, 0.5]	[−20.1, 8.7]	[18.1, 35.1]

Furthermore, for the $D_{max\;dose}^{diode}$, the range of cumulative dose differences for the three plans relative to the planned dose were [−3.0%, 1.1%] for the reference measurement, [−1.3%, 8.6%] for the moving but non‐gated measurement, and [−1.9%, 1.5%] for the gated measurement.

## 4 DISCUSSION

In this study, a novel MR‐compatible motion platform was assessed for use in the 1.5 T MR‐linac. The motion platform was thoroughly characterized using various metrics, with all measurements showing good agreement with the reference. During the measurements, it was found that for the evaluation of MR noise, grounding of the cable has significant influence on the noise measured. Therefore, it is recommended to carefully ground the cable connecting the control unit and the motion platform. The motion accuracy was shown to be high. Additionally, EPID measurements were more susceptible to noise, resulting in a larger standard deviation compared to the motor's self‐reported position. The influence of the motion platform on beam attenuation was found to be substantial. However, this does not pose an issue when performing dosimetric measurements involving beams going through the motion platform before reaching the detector. The impact of the motion platform can be accounted for by modeling it in the TPS, as was done here, showing a similar beam attenuation. This is commonly done when using QA devices to incorporate the influence of their geometry on dose measurements.

The motion platform's design offers a large surface suitable for accommodating various other phantoms. In this study a Delta4 phantom was mounted on the motion platform, illustrating the versatility in phantom selection. With a large load limit, the motion platform supports a wide range of heavy phantoms suitable for diverse QA and testing applications. Since the platform does not affect the MRI it is suitable for evaluation of MRI‐guided workflows for use with other phantoms containing dosimeters.

Measurements performed with the Delta4 showed the impact of motion and beam gating on the dose deposition during treatment. As expected, non‐gated measurements showed larger dosimetric deviations compared with the gated measurements. The benefit of this setup is that one can actually measure this difference as a function of time. This demonstrates a potential use case of this setup such as prospective patient‐specific plan QA using the patient's derived respiratory motion, and for instance evaluation of the impact of gating settings on the dose. This respiratory trace can be obtained from the CMM during a simulation, illustrating the real‐world dose delivery affected by motion, rather than measuring the planned, static dose. Additionally, this setup facilitates the assessment of effective margins and gating constraints. Using the Delta4 does come with the limitation that no complete online workflow can be performed, as the Delta4 cannot be scanned using MRI.

Several other motion platforms as for example the CIRS dynamic platform (Sun Nuclear, Melbourne, Florida, USA) and the ScandiDos Hexamotion (Uppsala, Sweden) are already commercially available, and can be used for QA purposes.^19^, ^20^ Both offer the ability to move a detector according to pre‐programmed motion trace, similar to the platform evaluated here. However, both the dynamic platform and the Hexamotion are not MR‐compatible, making these platforms unusable for evaluating workflows in the MR‐linac.

One limitation of the motion platform is its ability to move only in one direction. However, in the standard setup, the motion aligns with the cranial‐caudal direction,^21^ which is typically most affected by respiratory motion. This limitation can be partially addressed by rotating the motion platform (e.g., in the coronal or sagittal plane), allowing motion along two (or three) axes. Alternatively, combining the motion platform with a separate motion‐capable phantom could enable the simulation of movement in 2D or even 3D. Future research should be performed on the development and necessity of a platform which moves in 2D or even 3D.

## 5 CONCLUSION

All measurements performed with the IBA QUASAR Motion MR Platform on the 1.5 T MR‐linac showed that its effect on the MRI system and vice versa was negligible. Therefore, we conclude that the motion platform can be safely and effectively used in a 1.5 T magnetic field, to perform tasks such as motion‐included QA. This study aimed to evaluate the performance of the motion platform rather than develop QA methodologies for assessing motion compensation, which requires further research. The motion platform can support studies on motion‐compensated MR acquisition and real‐time adaptive treatment delivery using various phantoms.

## CONFLICT OF INTEREST STATEMENT

The authors declare no conflicts of interest.

## Supporting information

Supporting Information
