# Supplementary material for: Characterization of an MR‐compatible motion platform for quality assurance of motion‐compensated treatments on the 1.5 T MR‐linac
Source: Med Phys. 2025 Jan 31;52(5):3391–7. doi: 10.1002/mp.17632 (PMC12059540; doi:10.1002/mp.17632)
Supplement: Supplementary file 1 — Supporting Information [file MP-52-3391-s001.rtf]

1	Technical note: Characterization of an MR-compatible motion
2	platform for quality assurance of motion-compensated treatments on
3	the 1.5 T MR-linac


4  Stijn Oolbekkink1, Pim T.S. Borman1, Jochem W.H. Wolthaus1, Bram van Asselen1, Astrid
5  L.H.M.W. van Lier1, Stephanie Dunn2, Grant R. Koenig2, Nick Hartman2, Niusha Kheirkhah2, 
6  Bas W. Raaymakers1, Martin F. Fast1
7  1 Department of Radiotherapy, University Medical Center Utrecht, Heidelberglaan 100, 3584 CX,
8  Utrecht, the Netherlands
9  2 IBA QUASAR, Modus Medical Devices Inc., 1570 North Routledge Park, London, Ontario N6H
10  5L6, Canada

11  

12  S1. Scan parameters B0&B1 influence

Table 1: The scan parameter used for the B0&B1 measurements performed.

Protocol	FoV
(FH x RL x AP [mm]	Voxel [mm]	NSA	TE [ms]	TR [ms]	FA [deg]	
B0	450 x 450 x 105	2.0 x 2.0 x 5.0	4	5.4	65	20	
B1 - 60 deg	450 x 450 x 55	2.0 x 2.0 x 5.0	1	10	1000	60	
B1 - 120 deg	450 x 450 x 55	2.0 x 2.0 x 5.0	1	10	1000	120	
13  S2. ACR measurement results

14  All the results of the ACR measurements performed are shown in Tables 2 - 7. Standardized tests
15  using the American College of Radiologists (ACR) phantom were performed to assess the influence
16  of the (moving) motion platform on the geometric accuracy, percent-signal ghosting, image intensity
17  uniformity, slice position accuracy, slice thickness accuracy, and high-contrast spatial resolution
18  tests. If tolerances are applicable, these are shown in the tolerance row.

19  S2.1. Geometric accuracy

Table 2: The average results of the geometric accuracy test performed for the three setups.

	XY diameter
[mm]	Z length
[mm]	
Tolerance	190.0 ± 2.0 mm	148.0 ± 2.0 mm	
Reference	190.8	149.1	
Motion platform stationary	190.8	148.6	
Motion platform moving	190.8	149.5	

20  S2.2. Percent-signal ghosting and image intensity uniformity

Table 3: The average results of the percent-signal ghosting and image intensity uniformity tests performed for the three setups.

	Percent-signal
ghosting [%]	Percent integral uniformity [%]
T1	T2	
Tolerance	≤ 2.5%	≥ 87.5%	≥ 87.5%	
Reference	0.0	90.1	89.6	
Motion platform stationary	0.0	90.2	90.1	
Motion platform moving	0.0	89.9	90.2	
21  S2.3. Slice position accuracy

Table 4: The average results of the slice position accuracy test performed for the three setups.

	Slice position
error T1 [mm] Slice 1	Slice 11	Slice position
error T2 [mm] Slice 1	Slice 11	
Tolerance	± 5.0 mm	± 5.0 mm	± 5.0 mm	± 5.0 mm	
Reference	0.8	-2.9	0.7	-3.2	
Motion platform stationary	0.8	-2.8	0.6	-2.9	
Motion platform moving	0.8	-2.7	0.7	-3.2	
22  S2.4. Slice thickness accuracy

Table 5: The average results of the slice thickness accuracy test performed for the three setups.

Slice thickness [mm]
 T1	T2	
Tolerance	5.0 ± 0.7 mm	5.0 ± 0.7 mm	
Reference	5.0	4.9	
Motion platform stationary	4.9	4.6	
Motion platform moving	4.8	4.7	

23  S2.5. High-contrast spatial resolution
24  S2.5.1. T1 weighted image

Table 6: The average results of the T1 weighted high-contrast spatial resolution test performed for the three setups. If either of the two measurements was false, the measurement was considered a failure.
Resolution T1 hor.	Resolution T1 ver.
Resolution insert	0.9 mm	1.0 mm	1.1 mm	0.9 mm	1.0 mm	1.1 mm	
Reference	False	True	True	False	True	True	
Motion platform stationary	False	True	True	False	True	True	
Motion platform moving	False	True	True	False	True	True	
25  S2.5.2. T2 weighted image

Table 7: The average results of the T2 weighted high-contrast spatial resolution test performed for the three setups. If either of the two measurements was false, the measurement was considered a failure.
Resolution T2 hor.	Resolution T2 ver.
Resolution insert	0.9 mm	1.0 mm	1.1 mm	0.9 mm	1.0 mm	1.1 mm	
Reference	False	True	True	False	True	True	
Motion platform stationary	False	True	True	False	True	True	
Motion platform moving	False	True	True	False	True	True	
